# Outcome and prognostic variables in childhood rhabdomyosarcoma (RMS) with emphasis on impact of FOXO1 fusions in non-metastatic RMS: experience from a tertiary cancer centre in India

**DOI:** 10.3332/ecancer.2023.1539

**Published:** 2023-04-27

**Authors:** Subramaniam Ramanathan, Sneha Sisodiya, Omshree Shetty, Maya Prasad, Badira C Parambil, Sneha Shah, Mukta Ramadwar, Nehal Khanna, Siddhartha Laskar, Sajid Qureshi, Tushar Vora, Girish Chinnaswamy

**Affiliations:** 1 Trust Doctor, Department of Paediatric Oncology, Great North Children’s Hospital, Royal Victoria Infirmary, Newcastle-Upon-Tyne, NE1 8SG, UK; 2 Department of Pathology, Lokmanya Tilak Municipal General Hospital & Medical College, Mumbai 400022, India; 3 Division of Molecular Pathology, Department of Pathology, Tata Memorial Hospital, Mumbai 400012, India,; 4 Homi Bhabha National Institute, Mumbai 400094, India; 5 Department of Pediatric Oncology, Tata Memorial Hospital, Mumbai 400012, India; 6 Department of Nuclear Medicine, Tata Memorial Hospital, Mumbai 400012, India; 7 Department of Pathology, Tata Memorial Hospital, Mumbai 400012, India; 8 Department of Radiation Oncology, Tata Memorial Hospital, Mumbai 400012, India; 9 Department of Pediatric Surgery, Tata Memorial Hospital, Mumbai 400012, India; 10 Department of Pediatric Oncology, SickKids Hospital, Toronto ON M5G 1X8, Canada

**Keywords:** rhabdomyosarcoma, FOXO1 fusions, paediatric soft tissue sarcomas, LMIC, India

## Abstract

While factors influencing outcomes of rhabdomyosarcoma (RMS) in developed countries have evolved from clinical characteristics to molecular profiles, similar data from developing countries are scarce. This is a single-centre analysis of outcomes in treated cases of RMS, with emphasis on prevalence, risk-migration and prognostic impact of Forkhead Box O1 (FOXO1) in non-metastatic RMS. All children with histopathologically proven RMS, treated between January 2013 and December 2018 were included. Intergroup Rhabdomyosarcoma Study-4 risk stratification was used, with treatment based on a multimodality-regimen with chemotherapy (Vincristine/Ifosfamide/Etoposide and Vincristine/Actinomycin-D/Cyclophosphamide) and appropriate local therapy. Formalin-fixed paraffin-embedded tissues were tested using Reverse Transcriptase-Polymerase Chain Reaction for FOXO1-fusions (PAX3(P3F); PAX7(P7F)). A total of 221 children (Cohort-1) were included, of which 182 patients had non-metastatic disease (Cohort-2). Thirty-six (16%), 146 (66%), 39 (18%) patients were low-risk (LR), intermediate-risk (IR) and high-risk, respectively. FOXO1-fusion status was available in 140 patients with localised RMS (Cohort 3). P3F and P7F were detected in 25/49 (51%) and 14/85 (16.5%) of alveolar and embryonal variants, respectively. The 5-year-event-free survival (EFS)/overall survival (OS) of Cohorts 1, 2 and 3 was 48.5%/55.5%, 54.6%/62.6% and 55.1%/63.7%, respectively. Amongst the localised RMS, presence of nodal metastases and primary tumour size > 10 cms were adverse prognostic factorvs (*p* < 0.05). On incorporating fusion-status in risk-stratification, 6/29 (21%) patients migrated from LR (A/B) to IR. All patients who re-categorised as LR (FOXO1 negative) had a 5-year EFS/OS of 80.81%/90.91%. FOXO1-negative tumours had a better 5-year relapse-free survival (58.92% versus 44.63%; *p* = 0.296) with a near-significant correlation in favourable-site tumours (75.10% versus 45.83%; *p* = 0.063). While FOXO1-fusions have superior prognostic utility compared to histology alone in localised, favourable-site RMS, traditional prognostic factors (tumour size and nodal metastases) impacted outcome the most in this subset. Strengthening of early referral systems in community and timely local intervention can help in improving outcome in resource-constrained countries.

## Introduction

Rhabdomyosarcoma (RMS), the most common soft-tissue sarcoma, accounts for approximately 3.5% of childhood malignancies. With current therapy, localised disease has an over 70% 5-year event-free survival (EFS), and metastatic disease has survival rates varying from 5% to 38% [1]. Therapy in RMS is multidisciplinary, guided by site of the tumour, age of the child, extent of surgical resection, presence of distant metastases and histopathological subtype. Histopathologically, RMS is broadly divided into alveolar RMS (ARMS) and embryonal RMS (ERMS); ARMS was historically defined when any amount of biopsy component was alveolar with recent studies defining a threshold of ≥50% alveolar component to label ARMS [2]. This classification is vital as ARMS has been found to be associated with an inferior outcome when compared to its embryonal counterpart [3]. However, a biopsy may not be completely representative of the entire tumour. In addition, it is now known that ARMS lacking the characteristic Forkhead Box O1** (**FOXO1) fusion has a gene expression and clinical behaviour similar to ERMS [4]. Consequently, similar to other childhood solid tumours like neuroblastoma and medulloblastoma where molecular aberrations have made their way into frontline algorithms of therapy, PAX-FOXO1 fusion status (PAX3(P3F); PAX7(P7F)) has been incorporated in contemporary risk stratification in paediatric RMS [5, 6]. This retrospective study is an attempt at assessing the prevalence, risk-migration and prognostic impact of fusion status in localised childhood RMS, while also comparing the traditional prognostic variables like age, group, histological subtype, nodal status and tumour size in all treated cases of RMS at a single centre.

## Methodology

### Eligibility criteria

The study was a retrospective audit of all children under the age of 18 years with a histopathologically confirmed diagnosis of RMS, conducted over a 6-year period from January 2013 to December 2018. Children, in whom treatment records and molecular details were available, were considered eligible for analysis. Those who had received any kind of therapy in the form of chemotherapy or radiation prior to presentation to our hospital were excluded. Children who underwent biopsy or surgical excision outside, were staged accordingly and were considered eligible. Patients with metastatic disease who received treatment were also included.

### Staging and treatment

The stage, group and risk of the tumour were assigned as per the Children’s Oncology Group (COG) system of risk-stratification of childhood RMS [7]. Pre-treatment evaluation of all children included a complete blood count, serum electrolytes with biochemical parameters and coagulation profile. In our study cohort, majority of our patients underwent a positron emission tomography-computed tomography (PET-CT) scan at baseline to assess both locoregional spread and distant metastases. All patients underwent bilateral bone marrow aspiration and biopsy as a part of staging. Magnetic Resonance Imaging was an adjunct imaging modality in para-meningeal, para-spinal and genitourinary tumours. In addition, children with parameningeal tumours underwent cerebrospinal fluid cytology analysis. Semen cryopreservation was offered to young adolescent boys. Orbital tumours, tumours arising in head and neck region (non-parameningeal), genitourinary tumours (non-bladder, non-prostate, non-kidney) and biliary tree were considered ‘favourable site’ tumours and rest were ‘unfavourable site’. The tumour size was according to the largest dimension of the primary tumour reported on the pre-treatment imaging. Nodal involvement was defined as unequivocal clinico-radiological enlargement or nodal sampling [fine needle aspiration cytology (FNAC) or sampling].

**Figure 1.**
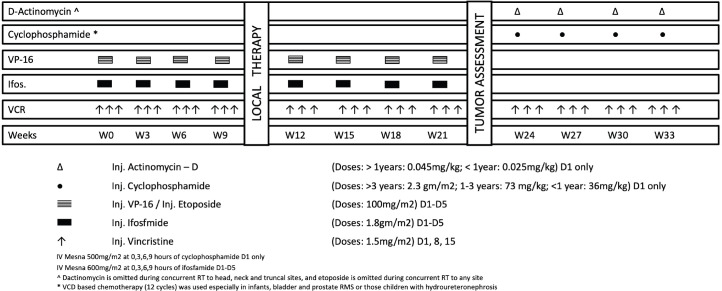
Chemotherapy protocol.

The study population was analysed as three cohorts: Cohort 1 consisted of all treated cases of RMS; Cohort 2 consisted of only localised RMS; Cohort 3 consisted of all localised cases with FOXO1 fusion details. The multi-agent combination chemotherapy is outlined in Figure 1. It comprised of eight cycles of Vincristine-Ifosfamide-Etoposide (cumulative ifosfamide dose: 72 gm/m^2^) and four cycles of Vincristine-Cyclophosphamide-Dactinomycin (cumulative cyclophosphamide dose: 8.8 gm/m^2^). Five patients (2.3%) in the low risk (LR) (A) subset received a truncated LR protocol comprising of Vincristine, Actinomycin-D and Cyclophosphamide (cumulative cyclophosphamide dose: 4.8 gm/m^2^) for 22 weeks [8]. After 9–12 weeks of neo-adjuvant chemotherapy, the choice and strategy for local control was finalised by a multidisciplinary planning meeting. The indications, timing and doses of radiation were administered as per institutional guidelines [9]. Following completion of treatment, children were followed up every 3 monthly in the first year, 6 monthly in the second year and yearly following that until the age of 5 years with chest radiograph at every visit and 6-monthly locoregional imaging of the primary site. Clinical examination of the primary site and evaluation of end-organ toxicity was performed at every follow-up visit.

### Histology and molecular testing

Tissue for histopathology was used for histological subtyping, immunohistochemistry [Desmin, Vimentin, MyoD1, Myogenin, S100, epithelial membrane antigen (EMA), leucocyte common antigen (LCA), fli1 and Mic2] and molecular subtyping. Alveolar morphology greater than 50% was categorised as alveolar subtype. Histology was ascertained after independent reporting by two trained pathologists (SSi and MR), Formalin fixed paraffin embedded tissue containing tumour tissue was used to perform reverse transcriptase-polymerase chain reaction (RT-PCR). The technique of RNA extraction, gel electrophoresis, results, equipment, reagents and materials used in the RT-PCR, PCR mix, PCR conditions are detailed in Appendix 1.

### Statistical analysis

The cohort was evaluated for both EFS and overall survival (OS). EFS was measured from the date of registration in the study until the date of the occurrence of the ﬁrst event, which was designated as relapse or progression or second malignancy or death. If no event occurred, then the date of the last follow-up was used as a censored observation. OS was measured from the date of registration in the study until the date of death. In surviving patients, the date of the last follow-up was used as a censored observation. For survival analysis, all patients were censored at the date of last follow-up or date of telephonic contact. EFS and OS were computed using Kaplan–Meier method. Statistical significance of possible prognostic factors was compared using log-rank test. Multivariate analysis using Cox proportional hazards model was performed to identify risk factors and a risk model. Stata 15.0 (June 2017) was used to compute all statistical data.

## Results

### Demographics

**Figure 2.**
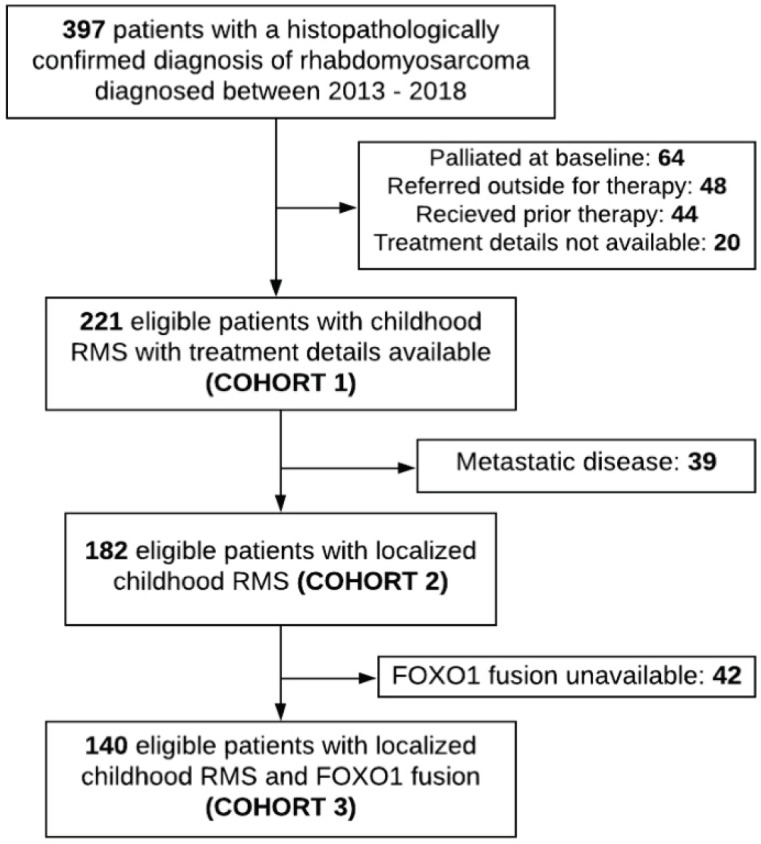
Flowchart of the patient profile.

The detailed profile of the eligible patients is summarised in Table 1. A total of 397 children were diagnosed with RMS during the study period. Of these, 64 (16.1%) patients were palliated because of disseminated disease (after a multi-disciplinary team meeting and parental choice), 48 (12%) were referred outside for therapy, 44 (11%) were pre-treated and treatment details were unavailable in 20 (5%). Two hundred and twenty-one children were considered eligible for the analysis (Figure 2) and regarded as Cohort 1. After exclusion of 39 patients (13%) with metastatic disease, 182 patients with localised disease formed Cohort 2. Of these, FOXO1 status was known in 140 patients which was designated as Cohort 3. The demographic variables and population characteristics are summarised in Table 1.

### Cohort distribution

Amongst the Cohort 1, lung was the commonest site of distant metastases (isolated-17; combination-3). Bone marrow metastases were found in 9 patients (isolated-2; combination-7). Other sites of metastases were distant lymph nodes outside the regional basin, bone and liver. One patient had adrenal metastases. Cohort 2 and Cohort 3 are similar in their distribution (Table 1). One hundred and forty children comprised Cohort 3 with a boy:girl ratio of 2.1:1 and a median age of 4.4 years (1.5–16.4 years). Tumours at unfavourable sites were found in a higher frequency in girls (80%; 36/45) when compared to boys (65.3%; 62/95) (*p* = 0.055). Majority of our patients were in between 1 and 9 years of age (*n*-109; 77.9%).

The subsequent description of results is focussed on patients comprising Cohort 3 (*n* = 140).

### FOXO1 fusions

Thirty-nine patients (27.9%) were FOXO1 positive, of which 27 (69.2%) were P3F and remaining were P7F (*n*-12; 30.8%). Forty-two patients (30%) had tumours at favourable sites, of which most common tumours are located in head and neck (non-parameningeal) region (*n*-20; 47.6%) followed by orbit (*n*-11; 26.2%), non-bladder/prostate genitourinary tract (*n*-10; 23.8%) and biliary tract (*n*-1; 2.4%). Amongst the tumours at unfavourable sites (*n*-98), parameningeal tumours were commonest (*n*-41; 41.8%) followed by extremities (*n*-30; 30.6%), genitourinary tract (*n*-8; 8.2%) and other sites like trunk, retroperitoneum and perineum (*n*-19; 19.4%). The distribution of FOXO1 positive tumours across favourable (12/42) and unfavourable (27/98) was similar (*p* = 0.90). Overall, ERMS and ARMS were noted in 85 (60.7%) and 49 (35%) cases, with FOXO1 fusion positive in 14 (16.4%) and 25 (51%) specimens, respectively. Of the remaining six cases, anaplastic and sclerosing morphologies were noted in three each. None of these six demonstrated FOXO1 positivity. P3F was twice as common as P7F in the whole cohort. While extremes of age, i.e. <1 year and >9 years had a nearly equal proportion of fusion negative and positive tumours, 25% patients in between 1 and 9 years were FOXO1 positive. Infant FOXO1 fusions were exclusively P3F.

**Figure 3.**
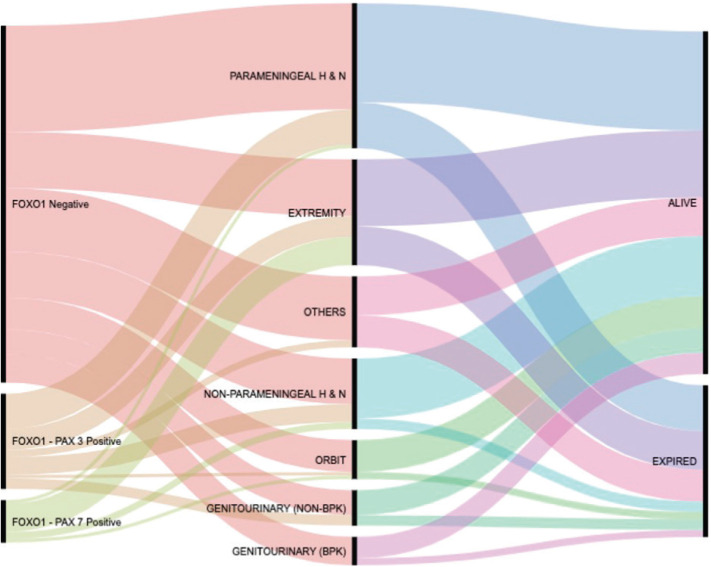
Alluvial plot of Cohort 3.

As per organ of origin, the frequency of P3F & P7F was found highest in extremity tumours (14/30; 46.7%) and lowest in orbital tumours (2/11; 18%). Figure 3 is an alluvial plot showing the spectrum of site distribution of paediatric RMS as per the FOXO1 fusion status and the eventual outcome (generated online on https://rawgraphs.io/learning) [10].

### Tumour size and nodal status

Sixty-six (47.1%) patients had tumours < 5 cm. Tumour size between 5 and 10 cms and >10 cms were seen in 57 (40.8%) and 17 (12.1%) patients, respectively. Majority of patients in larger tumours (tumours > 5 cms), size > 10 cm was found in 17/74 (23%) patients. Strikingly, majority of the tumours at favourable sites (79%) had a size < 5 cm when compared to tumours at unfavourable sites (34%; *p* < 0.001). Of the 17 patients with tumour size more than 10 cm, 14 (82%) were at unfavourable sites. FOXO1 positivity rates across different tumour sizes were equal (*p* = 0.544). Loco-regional nodal metastases were present in 53/140 patients. The proportion of nodal disease was comparable across FOXO1 fusion status (48.7% versus 33.6%; *p* = 0.10) and the site of the tumour (33.3% versus 39.8%; *p* = 0.47). Large tumours (> 10 cm) had a higher frequency of nodal metastases (70.5% versus 33.3%; *p* = 0.003).

### Risk stratification and risk migration

As per the institutional risk stratification (adopted from the COG staging and risk stratification system) [7], the number of patients in LR (A), LR (B) and intermediate risk (IR) was 15 (10.7%), 14 (10%) and 111 (79.3%), respectively. The FOXO1 positivity rates in these groups were 26%, 14.2% and 29.7%, respectively. The current ARTS1431 risk stratification, which employs FOXO1 status in lieu of histology has shifted LR (B) and FOXO1 positive tumours under IR tumours [5]. On applying these to our cohort, 18 patients migrated to IR. Based on FOXO1 fusions alone, 6/29 (20.7%) patients migrated to IR.

### Events

Thirty-five patients relapsed at a median time of 18.2 ± 7.5 months, of which local, metastatic and combined relapses were seen in 22 (62.8%), 9 (25.7%) and 4 (11.9%) patients, respectively. Three-fourths of the relapses (*n* = 28; 75%) occurred in patients with primary tumours at unfavourable sites and 12 (36%) amongst these were FOXO1 positive (P3F and P7F in 6 each). Amongst the relapses that occurred in favourable site – primary tumours (*n* = 7), 5 (71%) were FOXO1 positive (P3F: 3; P7F: 2). At last follow-up, 8/17 FOXO1+ve relapsed patients (47%) and 5/18 FOXO1-ve relapsed patients (28%) were noted to be alive (*p* = 0.2520). During therapy, 7 (5%) patients progressed, 8 (5.7%) patients abandoned therapy midway and 1 (0.7%) patient had a secondary acute myeloid leukaemia. None of the patients refused treatment. Forty patients died during the study period; the aetiologies being disease progression in 28 (70%), sepsis in 8 (20%), veno-occlusive disease and acute myeloid leukaemia in 1 (2.5%) each and unknown in 2 (5%) patients.

**Figure 4.**
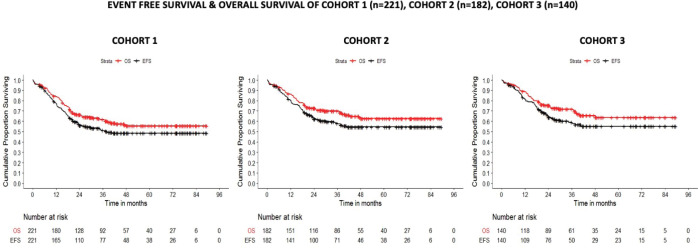
EFS and OS of all patients.

**Figure 5.**
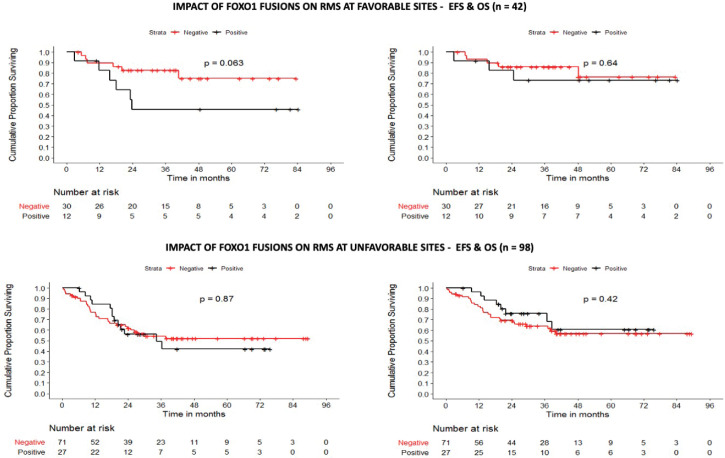
Impact of FOXO1 fusions on survival.

The 5-year EFS/OS of the Cohorts 1, 2 and 3 was 48.5%/55.5%, 54.6%/62.6% and 55.1%/63.7% at a median follow-up time of 36.4 ± 24.5, 39.2 ± 24.9 and 39.7 ± 22.9 months, respectively (Figure 4). In Cohort 1, the site of the primary tumour, tumour group, tumour size, nodal status and the revised risk stratification were found to be statistically significant variables affecting both EFS and OS. Amongst these, only nodal status (HR: 1.93) and tumour size >10 cms (HR: 2.24) retained their prognostic significance on multivariate analysis. Similarly, in localised tumours, tumour size and nodal status were noted to be statistically significant on both univariate and multivariate analysis. In patients with FOXO1 fusion data available (Cohort 3), while nodal status and site of primary tumour impacted EFS and OS on univariate analysis, primary tumour size was the strongest prognostic variable on both univariate and multivariate analysis impacting both EFS and OS (Table 2). On analysing the role of FOXO1 status, P3F and P7F independently failed to influence the EFS or OS. In the subset of favourable site tumours however, presence of FOXO1 fusions conferred an inferior survival (*p* = 0.063) (Figure 5). Of note, all patients who were re-categorised as LR (FOXO1 negative) had a 5-year OS of 91% (Table 2 and Table 3).

## Discussion

RMS is the commonest soft tissue sarcoma (STS) occurring in childhood. Factors influencing the outcome of RMS in developed countries have gradually evolved from clinical characteristics to molecular profiling but prognostic data on outcomes in RMS from developing countries are scarce. Ours is a large tertiary cancer centre and STSs form the commonest group of paediatric solid tumours, of which RMS is the most common histology (51%). A significant proportion of these patients often receive some form of treatment for symptom relief (improper surgical excision, alternative treatment) which is not cancer-directed. Because of this, they present with significant delay and with disseminated disease. In our cohort, a significant number (*n* = 68; 17%) with metastatic disease (to more than one site or bone/marrow metastases if single site) were palliated at presentation. This was a practical decision based on historically poor outcomes in this subset despite intensive therapy. It is also evident in this particular cohort, where amongst 39 patients with metastases who were treated (based on physician’s discretion and family preference), only 11 patients (28%) were alive at last follow-up. As the decision to treat metastatic patients has been fraught with biases and is therefore non-uniform, the discussion is focussed on outcomes of all children with localised RMS who received treatment at our centre. This approach renders comparability of uniformly treated patients under our care to that, available in literature.

In an attempt to refine risk-stratification, Hibbitts *et al* [6], in a recently published paper, provide a consolidated review of all the prognostic variables at play in childhood RMS, across six major COG trials (D9602, D9802, D9803, ARST00331, ARST0431 and ARST0531). Our cohort had a similar gender distribution and similar proportion of infantile RMS. In contrast, our cohort had nearly twice as many patients with nodal disease (37.9% versus 18%), higher proportion of large tumours (52.9% versus 45%), more tumours at unfavourable sites (70% versus 57%) and alveolar morphology (35% versus 25%). However, on comparing our cohort with regional studies, the demographic distribution of tumours was similar [11–13] (Table 4).

IR RMS is in itself a heterogenous rubric of patients with outcomes ranging between 60% and 83% for different subgroups as noted on the D9803 trial [14]. Application of FOXO1 fusion status is hence an effort to fine-tune the risk stratification and hopefully guide therapy in this relatively broad subset. Preliminary data regarding impact of FOXO1 fusions was conflicting. While the Cooperative Weichteilsarkom Studiengruppe (German Sarcoma Study Group) group (Germany) did not find any difference in the outcome with regard to FOXO1 status [15], the COG studies (America) clearly demonstrated an aggressive clinical phenotype in those harbouring FOXO1 fusions [4, 16]. Lack of association in the former study has been ascribed to the use of convenience cohorts (discrepancies between the outcomes of the patients whose tissue was available and not available), retrospective nature of analysis and differing treatment regimens [2].

In our cohort, the associations were strongest for traditional clinical variables like tumour size, nodal status and site of the tumour, with tumour size retaining its significance in multivariate analysis. Tumour size especially more than 10 cms had a clear adverse association with survival in our cohorts. FOXO1-positive tumours did have an inferior survival but this association was not significant on univariate analysis. In the subset of favourable tumours however, presence of FOXO1 fusions portended an inferior survival trending towards statistical significance (*p* = 0.063). The most recent COG publication collating data from six major trials (on 1,727 evaluable patients) has promulgated the adverse prognostic role of FOXO1 fusions as being second only to metastatic status and presently forms the cornerstone of current as well as future risk-stratification in childhood RMS [6]. Lack of such an emphatic association in our cohort can be explained by a smaller sample size. Several advantages exist on incorporating fusion studies with conventional histopathological reporting. The amount of alveolar component in a specimen to qualify it being called an ARMS continues to be a moving target [17], hence supplementing it with fusion studies allows for more objectivity. On applying the ARST1431 risk-stratification, we found that all patients (*n* = 11; Table 1) bracketed under the LR were surviving at the end of the study period. Aside from conventional prognostic factors and molecular profiles, there has also been interest in other factors like persistent disease metabolic activity demonstrated by PET-CT post definitive radiation being adversely associated with outcomes [18, 19].

The 5-year EFS and OS in our cohort is inferior to comparable cohorts from western studies. Contributing to inferior outcomes in low- and middle-income Countries (LMIC) are several issues like large tumours at baseline, advanced stage at presentation leading to upfront palliation, inconsistent use of local control modalities like radiation and high rate of treatment abandonment [20, 21]. While we have been able to curb the latter two by conducting regular multi-disciplinary meetings to ensure timely intervention and keeping in place a holistic patient tracking and support system [22], a streamlined approach of early referral of smaller/lower-stage tumours from the community is left wanting. Additionally, in LMIC setting, wherein triaging resources are important, knowledge on FOXO1 fusion helps guiding therapy and funnelling resources to the cohort that is expected to have a relatively better outcome. Being a retrospective study, the study has its limitations. A longer follow-up in these patients will shed more light on its impact on OS. Inability to offer therapy to all metastatic patients precludes identification of a subset within metastatic patients who would do better with the current approach. Therapy-wise, it was a pragmatic decision to offer a common chemotherapy backbone to all our patients, because majority of our patients belonged to IR and there was no convincing data that reducing therapy in the LR-subset would be a safe strategy in our patients. Molecular testing commenced in 2013, therefore a few patients escaped testing in the initial years. A more comprehensive testing of all paediatric RMS would elucidate the prognostic impact of FOXO1 fusion clearer. In addition, we did not test the presence of several non-FOXO1 PAX fusions like PAX-Nuclear receptor coactivator 2 (PAX-NCOA2) that may affect outcome adversely [23]. Despite these limitations, our study forms an important addition to prognostic impact of FOXO1 fusions in RMS; more so in a substantive cohort with larger tumours and higher incidence of nodal spread.

## Conclusions

Fusion studies have gradually moved on from being a desirable investigation in the work-up of RMS to an essential investigation. Streamlining risk-stratification using FOXO1 fusions has teased out a smaller yet highly favourable subset within the LR (A) and LR (B) RMS, who could be candidates for de-escalation of therapy. While clinical parameters continue to be an important component of treatment algorithms, addition of molecular markers to the risk stratification can help guide therapy. In conclusion, while FOXO1 fusion does make for an exciting addition in the armamentarium to RMS risk-stratification, other modifiable factors like strengthening of early referral systems and timely local intervention can help in improving outcomes in resource-constrained countries.

## List of abbreviations

ARMS, Alveolar rhabdomyosarcoma; COG, Children’s Oncology Group; EFS, Event-free survival; ERMS, Embryonal rhabdomyosarcoma; FOXO1, Forkhead Box O1; HR, High risk; IR, Intermediate risk; IRS, Intergroup rhabdomyosarcoma study; LMIC, Low- and middle-income countries; LR, Low risk; OS, Overall survival; PAX-FKHR, Paired box-forkhead in rhabdomyosarcoma; PET-CT, Positron Emission tomography-computed tomography; RMS, Rhabdomyosarcoma; RT-PCR, Reverse transcriptase-polymerase chain reaction.

## Author contributions

Subramaniam Ramanathan & Sneha Sisodiya contributed equally to the work.

## Conflicts of interest

The authors declare no conflicts of interest influencing the contents on this article.

## Financial disclosure

The authors have nothing to disclose.

## Data availability statement

Datasets generated during this study are available from the corresponding author on reasonable request.

## Figures and Tables

**Table 1.** Demographic and clinico-pathological characteristics of the study cohort.

Prognostic variable & categories	Cohort 1 (*n* = 221)		Cohort 2 (*n* = 182)			Cohort 3 (*n* = 140)										
						FOXO1 positive (n=39)							FOXO1 negative (*n* = 101)		Total	
						PAX3 (*n* = 27)				PAX7 (*n* = 12)						
**Age groups**	***n***	**%**	***n***	**%**		***n***	**%**			***n***	**%**		***n***	**%**	***n***	**%**
<10 years	174	78.70	148	81.30		17	63.00			11	91.60		87	86.10	115	82.20
≥10 years	47	21.30	34	18.70		10	37.00			01	8.40		14	13.90	25	17.80
**Group**																
Group 1	11	5.00	11	6.00		03	11.10			-	-		05	4.95	08	5.70
Group 2	05	2.30	05	2.70		01	3.80			-	-		02	2.00	03	2.10
Group 3	166	75.10	166	91.20		23	85.10			12	100		94	93.05	129	92.10
Group 4	39	17.60	NA			NA										
**Histology**																
Embryonal	142	64.30	118	64.80		07	26.00			07	58.34		71	70.30	85	60.70
Alveolar	67	30.30	55	30.20		20	74.00			05	41.66		24	23.70	49	35.00
Others	12	5.40	09	4.90		-	-			-	-		06	6.00	06	4.30
**Tumour size**																
<5 cm	90	40.70	80	44.00		16	59.20			04	33.33		46	45.50	66	47.10
5–10 cm	97	43.90	77	42.30		09	33.40			04	33.33		44	43.50	57	40.80
>10 cm	31	14.10	23	12.60		02	7.40			04	33.34		11	11.00	17	12.10
Insufficient data^a^	03	1.30	02	1.10		NA										
**Nodal status**																
N0	129	58.40	109	59.90		16	59.20			04	33.33		67	66.30	87	62.10
N1	90	40.70	71	39.00		11	40.80			08	66.67		34	33.70	53	37.90
Insufficient data^a^	02	0.90	02	1.10		NA										
**Site**																
Favourable	55	24.90	49	26.90		09	33.40			03	25.00		30	30.00	42	30.00
Unfavourable	166	75.10	133	73.10		18	66.60			09	75.00		71	70.00	98	70.00
**As per organ of origin**																
Orbit	12	5.40	12	6.60		01	3.80			01	8.30		09	8.90	11	7.90
Non-BPK genitourinary	20	9.00	15	8.20		03	11.10			-	-		07	6.90	10	7.10
Non-PM head & neck	22	10.00	21	11.50		05	18.50			02	16.70		13	12.90	20	14.30
PM head & neck	65	29.40	56	30.80		10	37.00			01	8.30		30	29.70	41	29.30
Genitourinary	18	8.10	16	8.80		-	-			-	-		08	8.00	08	5.70
Extremity	44	19.90	34	18.70		06	22.20			08	66.70		16	15.80	30	21.40
Others	40	18.10	28	15.40		02	7.40			-	-		18	17.80	20	14.30
**COG risk**																
LR (A)	20	9.00	20	11.00		03	11.10			01	8.40		11	10.90	15	10.70
LR (B)	16	7.20	16	8.80		01	3.80			01	8.30		12	11.90	14	10.00
IR	146	66.10	146	80.20		23	85.10			10	83.30		78	77.20	111	79.30
High risk	39	17.60	NA			NA										
**Revised risk^b^**																
LR	11	5.00	11	6.00		-	-			-	-		11	10.90	11	7.10
IR	166	75.10	166	91.20		27	100			12	100		90	89.10	129	92.90
High risk	39	17.60	NA													
Insufficient data^c^	05	2.30	05	2.80												
**FOXO1 fusion**																
PAX3 positive	33	14.90	27	14.80												
PAX7 positive	14	6.30	12	6.60												
Both negative	129	58.40	101	55.50												
Insufficient data	45	20.40	42	23.10												

a Data not available

b As per the ARST 1431 risk stratification

c Molecular data not available in 5 LR (A) patients

NA, Not applicable; PM, Parameningeal; BPK, Bladder/prostate/kidney

**Table 2.** Localised RMS with FOXO1 fusions.

Univariate analysis					
Prognostic variables & categories	EFS			OS	
	5 year (95% CI)	*p*-value		5 year (95% CI)	*p*-value
**Overall**	55.13% (45.55–63.70)	NA		63.68% (53.59–72.14)	NA
**Age**					
<10 years	44.63% (27.04–60.79)	0.198		63.65% (53.01–72.50)	0.407
≥10 years	58.92% (47.48–68.69)			62.52% (29.93–83.29)	
**Histology**					
Embryonal	54.58% (42.42–65.22)	0.946		59.68% (46.83–70.39)	0.238
Alveolar	56.04% (38.95–70.06)			65.31% (45.79–79.26)	
Others	53.33% (6.83–86.31)			100.00%	
**Fusion status**					
FOXO1 positive	44.63% (27.04–60.79)	0.296		65.89% (45.94–79.95)	0.603
FOXO1 negative	58.92% (47.48–68.69)			62.30% (49.86–72.49)	
**Site**					
Favourable	67.52% (49.09–80.51)	0.063		77.16% (58–88.39)	0.044
Unfavourable	53.05% (41.87–63.03)			58.00% (45.93–68.29)	
**Site & fusion**					
Favourable – FOXO1 positive	45.83% (16.89–71.02)	0.063		73.33% (37.90–90.56)	0.637
Favourable – FOXO1 negative	75.10% (50.24–88.77)			76.51% (47.48–90.82)	
Unfavourable – FOXO1 positive	42.10% (20.20–62.64)	0.807		60.74% (34.24–79.29)	0.661
Unfavourable – FOXO1 negative	52.06% (38.85–63.71)			56.76% (42.90–68.45)	
**Tumour size**					
<5 cm	71.59% (58.03–81.44)	<0.0001		81.94% (69.65–89.62)	<0.0001
5–10 cm	52.53% (37.30–65.68)			62.22% (45.92–74.88)	
>10 cm	0.00%			0.00%	
**Nodal status**					
N0	64.02% (51.98–73.78)	0.005		74.38% (62.37–83.07)	0.002
N1	40.33% (25.08–55.09)			45.73% (28.65–61.28)	
**Risk**					
Low (A)	72.22% (41.72–88.59)	0.45		86.67% (56.39–96.49)	0.25
Low (B)	45.08% (13.6–72.75)			52.6% (16.34–79.62)	
Intermediate	53.29% (42.48–62.97)			61.5% (50.13–71.01)	
**Revised risk**					
Low	80.81% (42.35–94.85)	0.127		90.91% (50.81–98.67)	0.105
Intermediate	52.61% (42.54–61.71)			61.12% (50.43–70.19)	
**Multivariate analysis**					
Covariate	Hazard ratio	p-value		Hazard ratio	p-value
Nodal status					
N0	Reference				
NI	1.58 (0.903–2.763)	0.109		1.803 (0.951–3.418)	0.071
**Tumour size**					
<5 cms	Reference				
5–10 cms	1.535 (0.790–2.983)	0.207		1.566 (0.703–3.505)	0.275
>10 cms	3.747 (1.741–8.062)	0.001		4.494 (1.878–10.754)	0.001
**Site**					
Favourable	Reference				
Unfavourable	1.424 (0.72–2.814)	0.31		1.635 (0.718–3.724)	0.242

**Table 3.** Prognostic variables influencing survival in all treated cases of RMS (5-year survival).

Univariate analysis									
Prognostic variables	All treated cases of childhood RMS; *n* = 221					All treated cases of localised RMS (L-RMS); *n* = 182			
	**5-year EFS (95% CI)**	***p*–value**	**5-year OS (95% CI)**	***p*–value**		**5-year EFS (95% CI)**	***p*–value**	**5-year OS (95% CI)**	***p*–value**
**Overall**	48.48% (41.28–55.31)	NA	55.46% (47.83–62.42)	NA		54.59% (46.49–61.97)	NA	62.61% (54.17–69.92)	NA
**Site**									
Favourable	66.52% (51.38–77.91)	<0.0001	73.63% (58.27–84.07)	<0.0001		72.97% (56.92–83.85)	0.004	81.12% (64.94–90.36)	0.004
Unfavourable	42.42% (34.27–50.33)		49.35% (40.54–57.56)			47.59% (38.16–56.42)		64.66% (55.63–72.32)	
**Age**									
<10 years	48.63% (40.55–56.21)	0.725	58.04% (49.82–65.39)	0.976		52.82% (43.91–60.96)	0.188	63.57% (54.68–71.18)	0.459
≥10 years	47.66% (31.26–62.36)		44.47% (25.95–61.46)			62.33% (41.47–77.57)		57.78% (33.52–75.93)	
**Histology**									
Embryonal	50.04% (41.13–58.29)	0.862	55.42% (46.28–63.62)	0.428		54.86% (44.95–63.72)	0.951	61.66% (51.60–70.24)	0.453
Alveolar	44.9% (31.54–57.37)		49.91% (34.06–63.86)			52.81% (37.02–66.35)		58.34% (39.75–73.00)	
Others	49.87% (17.29–75.90)		79.55% (39.32–94.54)			60.00% (19.55–85.23)		87.50% (38.70–98.14)	
**GROUP**									
Group I	80.81% (42.35–94.85)	<0.0001	90.00% (47.30–98.53)	<0.001		80.81% (42.35–94.85)	0.112	90% (47.3–98.53)	0.146
Group II	80% (20.38–96.92)		80.00% (20.38–96.92)			80.00% (20.38–96.92)		80.00% (20.38–96.92)	
Group III	51.87% (43.34–59.74)		60.15% (51.17–68.00)			51.87% (43.34–59.74)		60.15% (51.17–68.00)	
Group IV^c^	0.00%		0.00%			Not applicable			
**Tumour size**									
<5 cm	65.59% (53.83–75.03)	<0.0001	72.9% (60.71–81.85)	<0.0001		69.01% (56.43–78.63)	<0.0001	77.50% (64.57–86.20)	<0.0001
5–10 cm	44.02% (33.37–54.16)		53.28% (41.87–63.42)			53.99% (41.52–64.9)		64.26% (51.46–74.49)	
>10 cm	15.84% (4.97–32.25)		16.67% (3.91–34.65)			7.45% (0.65–26.25)		8.97% (0.80–30.00)	
**Nodal status**									
N0	61.02% (51.54-69.19)	<0.0001	69.15% (59.65–76.84)	<0.0001		65.2% (54.85–73.75)	0.003	74.45% (64.33–82.10)	0.002
N1	30.42% (20.37-41.06)		36.54% (25.33–47.80)			38.23% (25.82–50.52)		45.21% (31.51–57.95)	
Risk									
Low (A)	79.41% (53.97–91.74)	<0.0001	90.00% (65.60–97.40)	<0.0001		79.41% (53.97–91.74)	0.076	90.00% (65.60–97.40)	0.056
Low (B)	54.84% (24.04–77.64)		61.70% (28.24–83.16)			54.84% (24.04–77.64)		61.70% (28.24–83.16)	
Intermediate	50.50% (41.39–58.91)		58.49% (48.86–66.92)			50.5% (41.39–58.91)		58.49% (48.86–66.92)	
High^c^	0.00%		0.00%			Not applicable			
**Revised risk^a^**									
Low	80.81% (42.35–94.85)	<0.0001	90.91% (50.81–98.67)	<0.0001		80.81% (42.35–94.85)	0.051	90.91% (50.81–98.67)	0.066
Intermediate	51.08% (42.53–58.99)		59.33% (50.33–67.24)			51.08% (42.53–58.99)		59.33% (50.33–67.24)	
High^c^	0.00%		0.00%			Not applicable			
**Multivariate analysis**									
**Prognostic variable**	**Hazard ratio (95% CI)**	***p*-value**	**Hazard ratio (95% CI)**	***p*-value**		**Hazard ratio (95% CI)**	***p*-value**	**Hazard ratio (95% CI)**	***p*-value**
**Nodal status**									
N0	Reference					Reference			
N1	1.929 (1.29–2.88)	0.001	2.091 (1.339–3.264)	0.001		1.728 (1.064–2.806)	0.027	1.803 (1.039–3.127)	0.036
**Tumour size**									
<5 cms	Reference					Reference			
5–10 cms	1.581 (0.977–2.557)	0.062	1.521 (0.878–2.628)	0.133		1.417 (0.804–2.499)	0.228	1.394 (0.713–2.723)	0.332
>10 cms	2.239 (1.26–3.981)	0.006	2.581 (1.387–4.803)	0.003		3.651 (1.846–7.221)	< 0.001	4.422 (2.064–9.473)	<0.001
**Site**									
Favourable	Reference					Reference			
Unfavourable	1.291 (0.625–2.664)	0.49	2.428 (0.815–7.233)	0.111		1.72 (0.699–4.231)	0.238	1.808 (0.636–5.143)	0.267

a Only in patients with FOXO1 data available

b As per the ARST1431 COG risk stratification [5]

c 11 patients alive at last follow-up but not reached median time to follow-up

**Table 4.** Outcomes of RMS in similar studies from India.

Summary of recent studies in paediatric RMS from India^a^					
Variable	Bansal *et al* [13]	Dua *et al* [11]	Bhuvan *et al* [12]	Present study	
				Localised	All RMS
Sample size (years of accrual)	159 (23)	14 (6)	70 (16)	182 (6)	221 (6)
% distribution as per risk (LR/IR/HR)	35/56/11	28/50/22	18/52/30	20/80/-	9/7/66/18
% patients with nodal disease (N1)	47% (36/76)	Not specified	37%	39%	40%
% patients with large tumours (>5 cm)	68% (69/101)	Not specified	44%	56%	60%
% patients with tumours at unfavourable sites	67%	35%–50%	91%	73%	75%
% patients who received RT	66%	86%	55%	94%	95%
Dose of cyclophosphamide/cycle	Not specified	Not specified	1.8gm/m^2^	2.2 gm/m^2^	
FOXO1 fusion performed	No	No	No	Yes (140 localised RMS)	
EFS/OS^b^	5-year EFS: 43.6%	5-year EFS/OS: 57.1%/66.7%	3-year EFS/OS: 25.7%/49%	3-year EFS/OS: 58.00%/70.15% 5-year EFS/OS: 54.59%/62.61%	3-year EFS/OS: 51.03%/61.77% 5-year EFS/OS: 48.48%/55.46%
Abandonment rate	33%	14%	23%	5%	

a only full text papers included

b excluding abandonment

## Appendix 1. RNA extraction and reverse transcriptase-polymerase chain reaction (RT-PCR) analysis.

Total RNA was isolated from the FFPE tissue section using a Recover All Total Nucleic Acid Isolation kit (Ambion, USA). Extracted RNA was treated with RNase-free DNase I before cDNA preparation. cDNA was prepared using the H-Minus First strand cDNA synthesis system (Invitrogen). Briefly, 100 ng of total RNA was reverse transcribed into cDNA using random hexamers at 42°C for 1 hour followed by 72°C for 5 minutes. Two microlitres from the reaction was PCR amplified using PAX3 or PAX7 forward primer and FKHR reverse primer in a 20 μL reaction volume containing 10 pmol each of the forward and reverse primer, 10 μL 2× PCR master mix (Qiagen, Germany). To check the quality and integrity of the cDNA, ACTB was amplified as a housekeeping gene.

Results of molecular tests RT-PCR for PAX3-FKHR and PAX7-FKHR fusion studies were noted. Formalin-fixed paraffin-embedded tissue containing tumour tissue was used to perform RT-PCR. Equipment used in the RT-PCR, reagents and materials, PCR mix, PCR conditions is demonstrated as summarized below:

**Table A1.** List of equipment.

Name	Company	Catalogue no./model
Thermal cycler with heated lid	Applied biosystems	Veriti
Biosafety cabinet	Esco	AC2-3S1
Pipettes	Gilson	P2N,P20N,P200N,P100N
Bench top microcentrifuge	Eppendorf	5430

**Table A2.** List of reagents and materials used in RT-PCR.

Name	Company	Catalogue no.
Taq PCR Master-mix kit (2×)	Qiagen/ Thermo Scientific	201445/K0171
Nuclease – free water	-----	----
100 bp ladder	Fermentas	SM0241
Forward primer	Sigma Genosys	---
Reverse primer	Sigma Genosys	---
cDNA	---	---
Autoclaved H_2_O	----	---
0.2-μL thin-walled PCR tubes	Axygen	321-01-051
Sterile pipette tips	Tarsons	521000,521010
Cooled PCR rack	Tarsons	240060
Ice bucket plus ice	Sigma	Z407925
Glass bottle	Borosil	6254C
Powder-free gloves	Axygen	120323

All instruments were calibrated as per the standard guidelines. Positive control and negative control (Plasmid controls or known positive and negative cases) were used

### Procedure

Total RNA was isolated from the FFPE tissue section using a Recover All Total Nucleic Acid Isolation kit (Ambion, USA). Extracted RNA was treated with RNase-free DNase I before cDNA preparation. cDNA was prepared using the H-Minus First strand cDNA synthesis system (Invitrogen). Briefly, 100 ng of total RNA was reverse transcribed into cDNA using random hexamers at 42°C for 1 hour followed by 72°C for 5 minutes. Two microlitres from the reaction was PCR amplified using PAX3 or PAX7 forward primer and FKHR reverse primer in a 20 μL reaction volume containing 10 pmol each of the forward and reverse primer, 10 μL 2× PCR master mix (Qiagen, Germany). To check the quality and integrity of the cDNA, ACTB was amplified as a housekeeping gene.

The master mix was aliquot in all four tubes and the contents of the tube were properly mixed and placed in the thermal cycler.

PCR Primer sequences:

PAX3: 5’ TCC AAC CCC ATG AAC CCC 3’

FKHR: 5’ CTC TGG ATT GAG CAT CCA CC 3’

PAX7(P1): 5’ GAC AGC TTC ATG AAT CCG 3’

FKHR(P1): 5’ TTC CCG CTC TTG CCA CCC TCT GG 3’

**Table A3.** Preparation of PCR mix.

Reagents	Volume	Volume	Volume	Volume
2× Master mix	5 μL	5 μL	5 μL	5 μL
5 pM PAX3 primer (Forward)	0.5 μL	0.5 μL	0.5 μL	0.5 μL
5 pM FKHR primer (Reverse)	0.5 μL	0.5 μL	0.5 μL	0.5 μL
10 pM PAX7 primer (Forward)	0.5 μL	0.5 μL	0.5 μL	0.5 μL
10 pM FKHR primer (Reverse)	0.5 μL	0.5 μL	0.5 μL	0.5 μL
cDNA template	1 μL	--	--	--
Positive control	--	1 μL	--	--
Negative control	--	--	1 μL	--
H_2_O	3 μL	3 μL	3 μL	4 μL
Total	10 μL	10 μL	10 μL	10 μL
Sample no.	Test	Positive control	Negative control	Reagent control
Tube no.	1	2	3	4

**Table A4.** Required PCR conditions.

Cycle	Time	Events	Temp (°C)
1	5 minutes	Initial denaturation	94
35	30 seconds	Denaturation	94
	30 seconds	Annealing	66
	30 minutes	Extension	72
1	10 minutes	Final extension	72
Store	Forever		10

#### Gel electrophoresis

When the PCR was done, samples were run in 8%–10% polyacrylamide gel electrophoresis or 1.8% Agarose gel. They were stained with silver nitrate.

#### Sample loading in the gel

The test sample, positive, negative, and reagent control were loaded in the gel as shown in the figure below:

**Table:**

Lane 1	Lane 2	Lane 3	Lane 4	Lane 5	Lanes 6–10				
100 bp ladder	Test sample	Positive control	Negative control	Reagent control	-	-	-	-	-

#### Expected band size

PAX3-FKHR translocation: 141 bp

PAX7-FKHR translocation: 136 bp (nested)

The results were documented by taking a photograph of the gel using the gel electrophoresis unit.

### Results and interpretation

The results for PAX 3/PAX7-FKHR fusion were interpreted as follows:

#### Positive test

1) **PAX3: **141 bp band position – One band appearing in between 100 and 200 bp of molecular weight marker (Lane #1). Positive control also shows the band (Lane #2) and no bands are visible in the negative sample (Lanes #3 and #4).

2) **PAX7: **136 bp band position – One band appearing in between 100 and 200 bp of molecular weight marker (Lane #1). Positive control also shows the band (Lane #2). No bands were visible in the negative sample (Lanes #3 and #4).

#### Negative for PAX-FKHR

If there is no band in Lane #1 but positive control (Lane #2) shows band, was considered negative. PCR was repeated in case there were no bands in Lanes 3 1 and 2 or bands appeared in all the lanes.
